# Intake of the Total, Classes, and Subclasses of (Poly)Phenols and Risk of Prostate Cancer: A Prospective Analysis of the EPIC Study

**DOI:** 10.3390/cancers15164067

**Published:** 2023-08-11

**Authors:** Enrique Almanza-Aguilera, Daniel Guiñón-Fort, Aurora Perez-Cornago, Miriam Martínez-Huélamo, Cristina Andrés-Lacueva, Anne Tjønneland, Anne Kirstine Eriksen, Verena Katzke, Rashmita Bajracharya, Matthias B. Schulze, Giovanna Masala, Andreina Oliverio, Rosario Tumino, Luca Manfredi, Cristina Lasheras, Marta Crous-Bou, Maria-José Sánchez, Pilar Amiano, Sandra M. Colorado-Yohar, Marcela Guevara, Emily Sonestedt, Anders Bjartell, Elin Thysell, Elisabete Weiderpass, Dagfinn Aune, Elom K. Aglago, Ruth C. Travis, Raul Zamora-Ros

**Affiliations:** 1 Unit of Nutrition and Cancer, Cancer Epidemiology Research Program, Catalan Institute of Oncology (ICO), Bellvitge Biomedical Research Institute (IDIBELL), 08908 Barcelona, Spain; ealmanza@idibell.cat (E.A.-A.); dguinon@idibell.cat (D.G.-F.); marta.crous@iconcologia.net (M.C.-B.); 2 Cancer Epidemiology Unit, Nuffield Department of Population Health, University of Oxford, Oxford OX3 7LF, UK; aurora.perez-cornago@ndph.ox.ac.uk (A.P.-C.); ruth.travis@ndph.ox.ac.uk (R.C.T.); 3 Biomarkers and Nutrimetabolomics Laboratory, Department of Nutrition, Food Sciences and Gastronomy, Nutrition and Food Safety Research Institute (INSA), Food Innovation Network (XIA), Faculty of Pharmacy and Food Sciences, University of Barcelona, 08028 Barcelona, Spain; mmartinezh@ub.edu (M.M.-H.); candres@ub.edu (C.A.-L.); 4 Centro de Investigación Biomédica en Red de Fragilidad y Envejecimiento Saludable (CIBERFES), Instituto de Salud Carlos III, 28029 Madrid, Spain; 5 Danish Cancer Society Research Center, Danish Cancer Society, 2100 Copenhagen, Denmark; annet@cancer.dk (A.T.); ake@cancer.dk (A.K.E.); 6 Department of Public Health, University of Copenhagen, 2177 Copenhagen, Denmark; 7 Department of Cancer Epidemiology, German Cancer Research Center (DKFZ), 69120 Heidelberg, Germany; v.katzke@dkfz-heidelberg.de (V.K.); rashmita.bajracharya@dkfz-heidelberg.de (R.B.); 8 Department of Molecular Epidemiology, German Institute of Human Nutrition Potsdam-Rehbruecke, 14558 Nuthetal, Germany; mschulze@dife.de; 9 Institute of Nutritional Science, University of Potsdam, 14558 Nuthetal, Germany; 10 Cancer Risk Factors and Life-Style Epidemiology Unit, Institute for Cancer Research, Prevention and Clinical Network—ISPRO, 50139 Florence, Italy; g.masala@ispo.toscana.it; 11 Epidemiology and Prevention Unit, Department of Epidemiology and Data Science, Department of Research, Fondazione IRCCS Istituto Nazionale dei Tumori di Milano, 20133 Milan, Italy; andreina.oliverio@istitutotumori.mi.it; 12 Hyblean Association for Epidemiological Research (AIRE-ONLUS), 97100 Ragusa, Italy; rosario.tumino@asp.rg.it; 13 Department of Clinical and Biological Sciences, University of Turin, 10043 Orbassano, Italy; luca.manfredi@unito.it; 14 Functional Biology Department, University of Oviedo, 33003 Oviedo, Spain; lasheras@uniovi.es; 15 Granada Cancer Registry, Andalusian School of Public Health (EASP), Instituto de Investigación Biosanitaria Ibs. GRANADA, University of Granada, 18011 Granada, Spain; mariajose.sanchez.easp@juntadeandalucia.es; 16 Instituto de Investigación Biosanitaria Ibs. GRANADA, 18012 Granada, Spain; 17 Centro de Investigación Biomédica en Red de Epidemiología y Salud Pública (CIBERESP), 28029 Madrid, Spain; p-amiano@euskadi.eus (P.A.); scyohar@gmail.com (S.M.C.-Y.); mp.guevara.eslava@navarra.es (M.G.); 18 Department of Preventive Medicine and Public Health, University of Granada, 18071 Granada, Spain; 19 Ministry of Health of the Basque Government, Sub Directorate for Public Health and Addictions of Gipuzkoa, 20013 San Sebastian, Spain; 20 Epidemiology of Chronic and Communicable Diseases Group, BioGipuzkoa Health Research Institute, 20014 San Sebastian, Spain; 21 Department of Epidemiology, Murcia Regional Health Council, IMIB-Arrixaca, Murcia University, 30003 Murcia, Spain; 22 Research Group on Demography and Health, National Faculty of Public Health, University of Antioquia, Medellín 050010, Colombia; 23 Navarra Public Health Institute, 31003 Pamplona, Spain; 24 Navarra Institute for Health Research (IdiSNA), 31008 Pamplona, Spain; 25 Nutritional Epidemiology, Department of Clinical Sciences Malmö, Lund University, 214 28 Malmö, Sweden; emily.sonestedt@med.lu.se; 26 Department of Urology, Skåne University Hospital, 205 02 Malmö, Sweden; anders.bjartell@med.lu.se; 27 Department of Medical Biosciences, Umeå University, 901 87 Umeå, Sweden; elin.thysell@umu.se; 28 International Agency for Research on Cancer (IARC/WHO), 69372 Lyon, France; weiderpasse@iarc.who.int; 29 Department of Epidemiology and Biostatistics, School of Public Health, Imperial College London, London W2 1PG, UK; d.aune@imperial.ac.uk (D.A.); k.aglago@imperial.ac.uk (E.K.A.); 30 Department of Nutrition, Oslo New University College, 0456 Oslo, Norway; 31 Department of Endocrinology, Morbid Obesity and Preventive Medicine, Oslo University Hospital, 0424 Oslo, Norway

**Keywords:** polyphenols, diet, intake, prostate cancer, cohort, EPIC

## Abstract

**Simple Summary:**

(Poly)phenols are bioactive compounds naturally present in plant-based foods, but they have been suggested to increase the prostate cancer risk in retrospective case-control studies. Therefore, our aim was to prospectively evaluate these associations, including clinically relevant subtypes of prostate cancer. We investigated them using the European Prospective Investigation into Cancer and Nutrition (EPIC) cohort, a large observational study including 131,425 adult men from seven European countries. During 14 years of follow-up, a total of 6939 incident prostate cancer cases were identified. Overall, no statistically significant associations were observed between the baseline intake of any class and subclass of (poly)phenols and the risk of overall and any subtype of prostate cancer. In conclusion, the consumption of (poly)phenols and (poly)phenol-rich foods does not increase the risk of prostate cancer and can be included as part of a healthy diet.

**Abstract:**

Existing epidemiological evidence regarding the potential role of (poly)phenol intake in prostate cancer (PCa) risk is scarce and, in the case of flavonoids, it has been suggested that their intake may increase PCa risk. We investigated the associations between the intake of the total and individual classes and subclasses of (poly)phenols and the risk of PCa, including clinically relevant subtypes. The European Prospective Investigation into Cancer and Nutrition (EPIC) cohort included 131,425 adult men from seven European countries. (Poly)phenol intake at baseline was assessed by combining validated center/country-specific dietary questionnaires and the Phenol-Explorer database. Multivariable-adjusted Cox proportional hazards models were used to estimate the hazard ratios (HR) and 95% confidence intervals (CI). In total, 6939 incident PCa cases (including 3501 low-grade and 710 high-grade, 2446 localized and 1268 advanced, and 914 fatal Pca cases) were identified during a mean follow-up of 14 years. No associations were observed between the total intake of (poly)phenols and the risk of PCa, either overall (HR_log2_ = 0.99, 95% CI 0.94–1.04) or according to PCa subtype. Null associations were also found between all classes (phenolic acids, flavonoids, lignans, and stilbenes) and subclasses of (poly)phenol intake and the risk of PCa, overall and according to PCa subtype. The results of the current large prospective cohort study do not support any association between (poly)phenol intake and PCa incidence.

## 1. Introduction

Prostate cancer (PCa) is the second most frequent cancer and the fifth leading cause of cancer death in men worldwide, with over 1.4 million new cases and 375,000 deaths in 2020 [1]. Age, a family history of PCa, race, adult height, and a few hereditary syndromes are non-modifiable and well-established risk factors for PCa [2,3,4,5]. A number of dietary factors have been investigated in relation to prostate cancer risk, including red and processed meats, animal fat, dairy products, vegetables, fruits, tea, fish, and whole-grain products; while there is some suggestive evidence that a high intake of dairy products may be associated with a higher risk, no dietary factors have been convincingly shown to influence risk [2,5,6].

(Poly)phenols (synonym phenolic compounds) are a large family of bioactive compounds widely distributed in plants and plant-based foods such as fruits, vegetables, tea, coffee, wine, seeds, whole-grain cereals, and cocoa [7]. These compounds can be classified into four classes, namely flavonoids, phenolic acids, lignans, and stilbenes, and be subdivided in many sub-classes depending on the number of phenol units within their molecular structure, substituent groups, and/or the linkage type between phenol units [8]. The intake of (poly)phenols in European adults is largely heterogeneous, ranging from 584 mg/d in Greek women to 1786 mg/d in Danish men [9].

Experimental studies with cell lines and animal models have shown that (poly)phenols may act as chemopreventive agents in PCa due to their antioxidant and anti-inflammatory effects, and to their ability to modulate androgen receptors or their transactivation of signalling pathways, to induce cell cycle arrest and apoptosis, and to inhibit angiogenesis and metastasis [10]. However, evidence from epidemiological studies is still limited and inconclusive. For example, most published observational studies investigating the relationship between (poly)phenol intake and PCa risk have used a case–control design and/or have only focused on dietary flavonoids and lignans [11,12,13,14], leaving a longitudinal analysis of the relationship between the intake of other (poly)phenol subclasses and subsequent PCa risk yet unexplored. In addition, a recent meta-analysis estimated among cohort studies that the total dietary intake of flavonoids is positively associated with an increased risk of PCa [15].

Our aim in the current study was to examine the associations between the intake of total, classes, and subclasses of (poly)phenols and the risk of PCa overall, as well as the main PCa clinical subtypes in a large European population.

## 2. Materials and Methods

### 2.1. Study Population

The European Prospective Investigation into Cancer and Nutrition cohort (EPIC) study is an ongoing multicenter prospective cohort study aimed at evaluating the associations between dietary, lifestyle and genetic factors and cancer risk. The study enrolled over half a million subjects (including 153,457 men) from the general population between 1992 and 2000 at ages between 35 and 70 years. For the current analyses, we included male participants from Denmark, Germany, Italy, Spain, Sweden, The Netherlands, and the United Kingdom. Participants’ data from Greece were not available for the current study. Participants were excluded if they had either a prevalent cancer other than non-melanoma skin cancer at recruitment, had missing information on the date of diagnosis, had incomplete follow-up data, had missing non-dietary or dietary information, or had extreme energy intake and/or expenditure (i.e., participants in the higher and lower 1% of the distribution for the ratio between energy intakes to estimated energy requirement). After exclusions, a total of 131,425 men were included for analyses.

Ethical approval for the EPIC study was obtained from the ethical review board of the International Agency for Research on Cancer (IARC) and the local ethics committees in the participating countries. All participants gave written informed consent.

### 2.2. Follow-Up and Case Assessment

Primary incident PCa cancer cases were identified via record linkage with regional cancer registries in most of the centers, except in Germany, where follow-up was based on a combination of methods, including health insurance records, cancer and pathology registries, and an active follow-up evaluation of the study participants and their next-of-kin. The participants’ vital status was collected from regional or national mortality registries. PCa was defined as code C61 in the 10th Revision of the International Classification of Diseases (ICD10). Information on the grade (based on the Gleason grading system) and stage of PCa (based on the Tumor-Node-Metastasis classification system [TNM]) was collected from each center, when available. Grade was stratified as low-intermediate (Gleason score < 8, or grade coded as well, moderately, or poorly differentiated) or high grade (Gleason score ≥ 8, or grade coded as undifferentiated). The localized stage included those tumors confined within the prostate and with no metastasis at diagnosis (TNM staging score ≤ T2 and N0/Nx, and M0; or stage coded in the recruitment center as localized). Advanced cases included tumors that had spread beyond the prostate at diagnosis (T3-T4 and/or N1-N3 and/or M1, and/or stage coded at the recruitment center as metastatic). Fatal cases were those who died of PCa during the follow-up.

### 2.3. Dietary Collection

Information on the habitual diet of participants over the previous 12 months before recruitment was collected using validated country/center-specific dietary questionnaires [16]. Most centers utilized a self-administered food frequency questionnaire. In the remaining centers (Ragusa center in Italy, and all centers in Spain), participants were interviewed by trained staff members using a diet history questionnaire. In Malmö center (Sweden), a combination of a semi-quantitative food frequency questionnaire and a 7-day record was administered. The relative validity and reproducibility of these questionnaires was previously evaluated for food groups (including polyphenol-rich foods), macro- and micronutrients, but not for (poly)phenols specifically [17]. Daily food intakes were calculated in g/d. Alcohol (g/d), nutrients (g/d) and total energy (kcal/d) intakes were estimated using the standardized EPIC Nutrient Database [18].

Dietary (poly)phenols intakes (mg/d) were estimated using the Phenol-Explorer database [19], which contains content values for 502 polyphenols in 452 foods and beverages [20], together with retention factors for cooked and processed foods [9,21]. In the present study, the intakes of 483 individual (poly)phenols found in their natural form (mainly glucosides and esters) were estimated. These (poly)phenols were grouped into five major classes according to their chemical structure: flavonoids, phenolic acids, stilbenes, lignans, and other (poly)phenols. The intake of total (poly)phenols was calculated as the sum of all individual compounds.

### 2.4. Lifestyle Assessment

Information regarding participants’ lifestyle, such as smoking status, physical activity, education, and socioeconomic characteristics was obtained using standardized lifestyle questionnaires. Trained personnel from all centers took anthropometrics measurements, except for the Oxford center (UK), where participants reported their own anthropometrics measurements, and these were later validated [16,22].

### 2.5. Statistical Analysis

(Poly)phenol intake was analyzed as both a categorical and a continuous variable. For categorical analysis, (poly)phenol intake was divided into quintiles, according to the intake distribution among all participants. Tests for a linear trend were performed by assigning the medians of each quintile as scores. For the continuous distribution, the (poly)phenol intakes were log2-transformed to reduce the skewness of the original intake distributions. Thus, a one-unit increase in the log2 scale corresponds to a doubling in the intake. Cox proportional hazards models with age as the underlying time variable in all models were used to examine the associations between total, classes, and subclasses of (poly)phenol intakes and PCa risk. For each association, the hazard ratio (HR) and a 95% confidence interval (CI) were calculated. Age at recruitment served as the entry time, while age at diagnosis, death, or censoring date (whichever came first) served as the exit time. To ensure proportional hazards, Schoenfeld residuals were used for all models, revealing no evidence of the violation of the hypothesis. A total of three statistical models were performed. Model 1 was stratified by center and age at baseline (<40, from 41 to 75 in ranges of 5 years, and >75 years). Model 2 extended Model 1 by including variables such as smoking status (never, former, smoker, and not specified), level of physical activity (inactive, moderately inactive, moderately active, active, and not specified, according to the Cambridge index) [23], educational level (none, primary, technical or professional, secondary, longer or university, and not specified), marital status (single, together, and not specified), self-reported diabetes prevalence (yes, not, and not specified), alcohol intake (0.0, >0–5.0, 5.0–14.9, 15.0–29.9, and >30.0 g/d), BMI (<22.5, 22.5–24.9, 25.0–29.9, and >30.0 kg/m^2^) and total energy intake (kcal/d). Model 3 was further adjusted for intake of total fiber (g/d) and vitamin C (g/d). All covariates included in the models were based on a priori assumptions.

Possible interactions between the intake of (poly)phenols (i.e., total, classes, subclasses of (poly)phenols) and the following covariates were examined by including an interaction term in the multi-adjusted models: age (<65 and >65 years), BMI (<25.0, 25–29.9 kg/m^2^, and >30.0 kg/m^2^), and smoking status (never, former or current smokers, and not specified). A likelihood ratio test was used in order to evaluate the significance of the interactions on a multiplicative scale. To fully assess the risk of PCa, we defined separate models according to clinically relevant subtypes: low- and high-grade, localized and advanced, and fatal PCa, and evaluated the heterogeneity between them using the Wald test. Sensitivity analyses were conducted, excluding 275 and 982 total PCa cases diagnosed during the first 2 and 5 years of follow-up, respectively. Statistical significance was attributed to results with *p*-value < 0.05, and *p*-value < 0.002 after Bonferroni correction (i.e., <0.05/23, the number of tests for the intakes of all (poly)phenol classes and subclasses) to account for multiple comparisons. Statistical analyses were carried out using R (version 4.2.1) and RStudio (version 2022.07.1) software.

## 3. Results

Overall, 6939 out of 131,425 men included in this study were diagnosed with a primary malignant PCa during a mean (SD) of 14 (SD: 4.7) years of follow-up (Table 1). According to the stage and grade of the disease, PCa cases were mostly localized (*n* = 2446) and low-grade (*n* = 3501), representing 35.3% and 50.5% of the total number of PCa cases, respectively. A total of 914 PCa cases (13.2%) resulted in a fatal outcome. The median (10th–90th percentiles) intake of total (poly)phenols among all participants was 1167 (625–1931) mg/d. The highest median intake of total (poly)phenols was observed in Denmark (1593 mg/d) and the lowest in Spain (834 mg/d) (Table 1). Men in the highest quintile of total (poly)phenol intake were older, more physically active, less likely to have diabetes, but more likely to smoke and less likely to be married, had a lower BMI and had a higher education level compared to those in the lowest quintile (Table 2). Furthermore, men with higher total (poly)phenol intakes consumed more total energy, alcohol, fiber, and vitamin C compared to those with lower intakes (Table 2). There was no difference in the baseline characteristics between PCa cases with and without stage or grade data, except that cases without grade or stage data were slightly older than those with these data (Supplementary Table S1).

No statistically significant association was observed between the intake of total (poly)phenols and the overall risk of PCa, using either the comparison between extreme quintiles (HR_Q5vs.Q1_ = 1.02; 95% CI 0.92–1.13; *p*-trend = 0.77) or the continuous distribution (HR_log2_ = 0.99; 95% CI 0.94–1.04) (Table 3). There was no evidence of significant heterogeneity in these associations according to PCa clinical subtype, except for small differences between associations with localized and advanced PCa for the intake of flavones and alkylphenols (Table 4). None of the classes and subclasses of (poly)phenols were associated with the risk of either overall PCa (Table 3) or the PCa clinical subtype, or fatal PCa (Table 4).

There were no statistically significant interactions between total (poly)phenol intake and overall PCa risk in the multivariable model according to either age at recruitment (*p* for interaction = 0.37) or smoking status (*p* for interaction = 0.74). A borderline interaction between the BMI categories and the total (poly)phenols (*p* for interaction = 0.05) in relation to the overall PCa risk was found. After dividing the results according to the BMI categories, no associations were observed between the intake of the main classes of (poly)phenols and PCa risk (Supplementary Table S2). Similarly, null results were observed after the exclusion of 275 or 982 PCa cases diagnosed in the first 2 and 5 years of follow-up, respectively.

## 4. Discussion

In this large prospective European study including more than 130,000 men and almost 7000 incident PCa cases, we found that the pre-diagnostic intake of total, classes, and subclasses of (poly)phenols was not associated with PCa risk, including with tumor subtypes, and PCa mortality.

To the best of our knowledge, this is the first study evaluating the prospective associations between the intake of total (poly)phenols, phenolic acids, stilbenes and other minor subclasses of (poly)phenols such as tyrosols, alkylphenols, and alkylmethoxyphenols, and PCa risk. So far, the only previous epidemiological evidence on the intake of total (poly)phenols, phenolic acids, and stilbenes and PCa risk comes from two case-control studies [24,25]. The study performed by Ghanavati et al., which included 97 PCa cases and 205 hospital-based controls, showed a significant inverse relationship between the high intake of total (poly)phenols, phenolic acids, and stilbenes, and the risk of PCa [24]. The study performed by Russo et al. (118 PCa cases and 222 controls) showed that although the intake of total phenolic acids was not associated with PCa risk, the intakes of caffeic acid and ferulic acid were inversely associated with overall PCa risk, and the higher intakes of hydroxybenzoic and caffeic acids were associated with a lower risk of advanced PCa [25].

For flavonoids, in contrast to our null findings, the results from a recent meta-analysis of three prospective cohort studies showed an increased risk of PCa according to higher intakes of total flavonoids (OR = 1.11; 95% CI 1.01–1.22) [15]. Notably, this association was shown to be consistent even after pooling the results with data from three case-control studies. In addition, in the same meta-analysis, a subgroup analysis stratified according to flavonoids subclasses showed that higher intakes of anthocyanidins and flavan-3-ols were significantly associated with increased PCa risk in two prospective studies [15]. Differences in the size and characteristics of participants, as well as methodological aspects related to both the collection and estimation of dietary intake, may explain the differences in the associations found in our study and those reported in the meta-analysis of Liu et al. For example, the size of our population was not only larger in its total number of participants, but also in its number of PCa cases. Furthermore, we included participants from seven European countries, while the cohorts analyzed by Liu et al. were only from Finland [26,27] and the USA [28]. Finally, while we used the Phenol-Explorer database to make flavonoids intake estimations, the cohort studies included in the meta-analysis of Liu et al. used a Finnish [26] and the USDA [27,28] databases for the flavonoid content of selected foods. A few differences for some flavonoid subclasses were observed in a study comparing the degree of reliability among flavonoid intakes estimated using Phenol-Explorer and the USDA databases [29]; therefore, caution is recommended when comparing the results using both databases.

In the present study, we did not observe any significant association between the total dietary intakes of isoflavones and lignans, and the total and clinical subtypes of PCa risk. Regarding isoflavones, our results are in accordance with those reported in the meta-analyses of Liu et al. [15], who in a pooled analysis of three prospective cohort studies, showed that the intake of total isoflavones was not associated with the risk of PCa. This result was similar when data from five case-control were added to the analysis in the same meta-analysis. To the best of our knowledge, there are no previous cohort studies investigating the relationship between the intake of lignans and PCa risk. Instead, evidence is limited to case-control studies, and the results from a meta-analysis by He et al. of three case-control studies suggested that the intake of total lignans was not associated with the PCa risk [30].

The mainly null associations between (poly)phenol intake and PCa reported in previous prospective studies are similar to those found for its main dietary sources: coffee, tea, fruits, and vegetables. In the EPIC study, Sen et al. found no evidence of an association between coffee or tea consumption and the risk of total and clinical subtypes of PCa [31]. The results related to coffee were similar to those later reported in other large-scale cohorts [32,33,34], but opposite to those estimated in a recent meta-analysis of 16 prospective cohort studies showing that a higher coffee consumption is significantly associated with a lower risk of PCa [35]. It should be noted, however, that the latter cohort studies [32,33,34] confirming the results of Sen et al. in 2019 were not included in this meta-analysis because they were published after the research period. Regarding tea, the findings reported by Sen et al. in the EPIC study were consistent with the pooled analysis of nine (i.e., eight prospective and one retrospective) and five (prospective) cohort studies in two meta-analyses, respectively [36,37]. Both meta-analyses (which included also eighteen and eight case-control studies, respectively) showed that among the cohort studies, there was a null association between the intakes of total, black or green tea and PCa risk. In a previous EPIC sub-study, Perez-Cornago found that a higher consumption of total fruits, but not total vegetables, was associated with a reduction in PCa risk [38]. However, this result was not supported by a recent meta-analysis that, by pooling data from 17 prospective cohort studies, reported an insignificant relationship between the intake of total fruits and vegetables and the risk of PCa [39].

The differences between the prospective epidemiological evidence and the findings from some experimental studies, which have suggested that the intake of certain (poly)phenols reduces the risk and progression of PCa [10,40], may be due to differences in several aspects, including exposure and outcome assessments, the doses or concentrations administered/consumed, and the duration of the study [39]. In addition, native (poly)phenols are used in cell culture studies, while (poly)phenols probably act via their metabolites rather than via the parent compounds in the human body [10]. In recent years, it has been suggested that in observational studies, the direct measurement of (poly)phenols and their metabolites via biomarkers in biological samples such as blood may not only improve the estimation of (poly)phenols exposure, but also the assessment of their possible protective effects on cancer development [41,42,43,44]. To date, however, studies using such approach to assess the prospective association between the biomarkers of (poly)phenols and PCa risk have mostly been either of a relatively small size, focused on selected (poly)phenols (i.e., isoflavones and lignans), or have led to inconclusive results [44,45,46,47,48,49].

Our study has several strengths and limitations. Its strengths include it being a prospective design, having a large sample size, a long follow-up, and the coverage of several European countries with large dietary heterogeneity. The limitations of these analyses include potential errors in dietary intake assessments that could lead to the attenuation of any true association between the intake of (poly)phenols and PCa risk. Specifically, self-reported dietary questionnaires may introduce bias into the (poly)phenol intake assessment as a result of random and systematic measurement errors, although the questionnaires were validated in each center/country. Regarding Phenol-Explorer, although it is the most comprehensive food composition database on (poly)phenols to date, it has limitations in covering all foods and the variability of PP content in foods, which could contribute to an underestimation of (poly)phenols intake. Furthermore, data on diet and lifestyle were only evaluated at baseline, thus potential changes in these variables during the follow-up were not accounted for in the models. In this sense, some individuals may have modified their diet during the early pre-diagnostic period of PCa; however, sensitivity analyses excluding incident PCa cases diagnosed in either the first 2 or 5 years of follow-up did not significantly alter the risk estimates. Finally, although we controlled for a wide range of established PCa risk factors, the possibility of residual confounding cannot be ruled out.

## 5. Conclusions

In conclusion, no associations were observed between the intake of the total, classes, and subclasses of (poly)phenols and the risk of PCa and its main clinically relevant subtypes in this large multi-center European cohort. Of note, our results do not support a previous meta-analysis showing that, among cohort studies, the total intake of flavonoids is related to a higher PCa risk [15].

## Figures and Tables

**Table 1:** Number of participants, prostate cancer cases and amount of (poly)phenol intake according to EPIC countries.

Country	*n*	Overall PCa	PCa Grade		PCa Stage		Fatal PCa	Total (Poly)Phenol Intake (mg/d) Median (P10–P90)
			Low-Grade	High-Grade	Localized	Advanced		
Sweden	22,306	1833	476	79	556	86	224	887 (536–1375)
Denmark	26,294	1885	652	240	540	550	312	1593 (965–2236)
The Netherlands	9627	215	189	17	32	74	22	1154 (719–1662)
Germany	21,178	833	687	68	533	186	45	1093 (652–1778)
United Kingdom	22,849	1028	636	183	258	206	217	1508 (917–2108)
Spain	15,139	666	527	77	434	74	61	834 (418–1482)
Italy	14,032	479	334	46	93	92	33	1008 (615–1517)
Total	131,425	6939	3501	710	2446	1268	914	1167 (625–1931)

Abbreviations: PCa, prostate cancer; P10, 10th percentile; P90, 90th percentile. Missing data for PCa grade = 2728 (39.3%); for PCa stage = 3225 (46.5%).

**Table 2:** Baseline characteristics of participants according to quintiles of total (poly)phenol intake in the EPIC cohort.

Baseline Characteristics	Quintiles of Total (Poly)phenol Intake					Total
	Quintile 1	Quintile 2	Quintile 3	Quintile 4	Quintile 5	
N	26,285	26,285	26,285	26,285	26,285	131,425
Cut-off (poly)phenol intake (mg/d)	<783	783–1040	1040–1310	1310–1662	>1662	
	Mean (SD)	Mean (SD)	Mean (SD)	Mean (SD)	Mean (SD)	Mean (SD)
Age at recruitment (years)	51.4 (10.0)	51.2 (10.0)	51.6 (10.1)	52.9 (10.0)	53.8 (9.20)	52.2 (9.90)
Total energy intake (kcal)	2134 (603)	2318 (601)	2445 (633)	2512 (658)	2675 (681)	2417 (662)
Fiber intake (mg/d)	20.5 (7.31)	22.7 (7.40)	24.3 (7.71)	25.5 (8.20)	28.6 (9.40)	24.3 (8.50)
Vitamin C intake (mg/d)	97.9 (55.4)	108 (59.3)	115 (60.1)	120 (62.2)	128 (70.2)	114.3 (62.5)
Smoking status	n (%)	n (%)	n (%)	n (%)	n (%)	n (%)
Never	10,055 (38.3%)	9491 (36.1%)	8879 (33.8%)	8297 (31.6%)	7488 (28.5%)	44,210 (33.6%)
Former	8969 (34.1%)	9610 (36.6%)	9931 (37.8%)	10,017 (38.1%)	9753 (37.1%)	48,280 (36.7%)
Current	7019 (26.7%)	6970 (26.5%)	7229 (27.5%)	7644 (29.1%)	8690 (33.1%)	37,552 (28.6%)
Not specified	242 (0.90%)	214 (0.80%)	246 (0.90%)	327 (1.20%)	354 (1.30%)	1383 (1.10%)
Physical activity level						
Inactive	5254 (20.0%)	4733 (18.0%)	4348 (16.5%)	4509 (17.2%)	4231 (16.1%)	23,075 (17.6%)
Moderately inactive	8614 (32.8%)	8470 (32.2%)	8187 (31.1%)	7922 (30.1%)	7453 (28.4%)	40,646 (30.9%)
Moderately active	6571 (25.0%)	6475 (24.6%)	6384 (24.3%)	6054 (23.0%)	6195 (23.6%)	31,679 (24.1%)
Active	5488 (20.9%)	6041 (23.0%)	6551 (24.9%)	6984 (26.6%)	7888 (30.0%)	32,952 (25.1%)
Not specified	358 (1.4%)	566 (2.2%)	815 (3.1%)	816 (3.1%)	518 (2.0%)	3073 (2.3%)
Educational level						
None	2048 (7.8%)	896 (3.4%)	638 (2.4%)	426 (1.6%)	257 (1.0%)	4265 (3.2%)
Primary	8987 (34.2%)	7910 (30.1%)	7067 (26.9%)	6794 (25.8%)	6942 (26.4%)	37,700 (28.7%)
Technical/Professional	5468 (20.8%)	6205 (23.6%)	6774 (25.8%)	7094 (27.0%)	7118 (27.1%)	32,659 (24.8%)
Secondary	3932 (15.0%)	4208 (16.0%)	3779 (14.4%)	3022 (11.5%)	2508 (9.5%)	17,449 (13.3%)
Longer (University)	5515 (21.0%)	6651 (25.3%)	7326 (27.9%)	7822 (29.8%)	8208 (31.2%)	35,522 (27.0%)
Not specified	335 (1.3%)	415 (1.6%)	701 (2.7%)	1127 (4.3%)	1252 (4.8%)	3830 (2.9%)
Marital status						
Single	4171 (15.9%)	3922 (14.9%)	3758 (14.3%)	3224 (12.3%)	2574 (9.80%)	17,649 (13.4%)
Together	13,747 (52.3%)	16,051 (61.1%)	15,429 (58.7%)	13,887 (52.8%)	10,705 (40.7%)	69,819 (53.1%)
Not specified	8367 (31.8%)	6312 (24.0%)	7098 (27.0%)	9174 (34.9%)	13,006 (49.5%)	43,957 (33.4%)
Diabetes prevalence						
No	24,325 (92.5%)	24,106 (91.7%)	23,175 (88.2%)	21,403 (81.4%)	20,760 (79.0%)	113,769 (86.6%)
Yes	1088 (4.1%)	898 (3.4%)	799 (3.0%)	777 (3.0%)	770 (2.9%)	4332 (3.3%)
Not specified	872 (3.3%)	1281 (4.9%)	2311 (8.8%)	4105 (15.6%)	4755 (18.1%)	13,324 (10.1%)
Alcohol intake (g/d)						
0.0	2860 (10.9%)	1487 (5.7%)	1289 (4.9%)	1278 (4.9%)	1186 (4.5%)	8100 (6.2%)
>0.0–< 5.0	7763 (29.5%)	6487 (24.7%)	5244 (20.0%)	4828 (18.4%)	4536 (17.3%)	28,858 (22.0%)
5.0–14.9	6848 (26.1%)	7263 (27.6%)	6891 (26.2%)	6835 (26.0%)	7022 (26.7%)	34,859 (26.5%)
15.0–29.9	4658 (17.7%)	5713 (21.7%)	5927 (22.5%)	5528 (21.0%)	5330 (20.3%)	27,156 (20.7%)
≥30.0	4156 (15.8%)	5335 (20.3%)	6934 (26.4%)	7816 (29.7%)	8211 (31.2%)	32,452 (24.7%)
Body Mass Index (kg/m^2^)						
<22.5	2941 (11.2%)	3134 (11.9%)	3244 (12.3%)	3292 (12.5%)	3400 (12.9%)	16,011 (12.2%)
≥22.5–24.9	5731 (21.8%)	6402 (24.4%)	6611 (25.2%)	6822 (26.0%)	7126 (27.1%)	32,692 (24.8%)
≥25.0–29.9	13,015 (49.5%)	12,849 (48.9%)	12,780 (48.6%)	12,613 (48.0%)	12,453 (47.4%)	63,710 (48.5%)
≥30.0	4598 (17.5%)	9536 (36.3%)	3650 (13.9%)	3558 (13.5%)	3306 (12.6%)	19,012 (14.4%)

**Table 3:** Hazard ratios (CI 95%) for total prostate cancer cases, according to quintiles of the intake of total, classes, and subclasses of (poly)phenols in the EPIC cohort.

	Intake (mg/d) Median (P10–P90)	Quintile 1 HR (95% CI)	Quintile 2 HR (95% CI)	Quintile 3 HR (95% CI)	Quintile 4 HR (95% CI)	Quintile 5 HR (95% CI)	P-Trend	Continuous (log2) HR (95% CI)
Total (poly)phenols	1167 (625–1931)	1.00 (ref)	1.10 (1.02–1.19)	1.02 (0.93–1.11)	1.02 (0.93–1.12)	1.02 (0.92–1.13)	0.77	0.99 (0.94–1.04)
Flavonoids	437 (159–1063)	1.00 (ref)	0.99 (0.92–1.07)	1.01 (0.94–1.10)	1.03 (0.94–1.12)	0.97 (0.88–1.07)	0.63	1.20 (0.98–1.04)
Flavanols	302 (96.1–853)	1.00 (ref)	0.99 (0.92–1.07)	1.00 (0.92–1.08)	1.03 (0.95–1.12)	0.94 (0.85–1.03)	0.23	1.01 (0.99–1.04)
Flavan-3-ol monomers	48.5 (11.6–430)	1.00 (ref)	1.01 (0.94–1.09)	1.02 (0.94–1.11)	1.03 (0.94–1.12)	1.02 (0.93–1.11)	0.89	1.01 (0.99–1.02)
Proanthocyanidins	211 (74.3–468)	1.00 (ref)	1.03 (0.96–1.11)	1.04 (0.96–1.13)	1.02 (0.94–1.12)	1.03 (0.94–1.14)	0.66	1.01 (1.00–1.02)
Theaflavins	2.05 (0.00–99.6)	1.00 (ref)	1.08 (0.96–1.22)	1.08 (0.98–1.18)	1.09 (1.00–1.20)	1.03 (0.94–1.14)	0.57	1.00 (1.00–1.01)
Flavonols	29.0 (10.2–93.8)	1.00 (ref)	0.94 (0.87–1.02)	1.05 (0.97–1.14)	1.01 (0.92–1.11)	1.01 (0.92–1.12)	0.62	1.01 (0.98–1.03)
Flavanones	22.5 (3.98–86.1)	1.00 (ref)	0.95 (0.88–1.02)	0.97 (0.90–1.05)	0.98 (0.90–1.06)	0.97 (0.88–1.07)	0.81	0.99 (0.98–1.01)
Anthocyanins	22.1 (4.87–90.1)	1.00 (ref)	1.03 (0.96–1.12)	1.05 (0.97–1.14)	1.09 (1.00–1.18)	1.02 (0.93–1.12)	0.87	1.01 (1.00–1.03)
Flavones	9.00 (3.15–23.1)	1.00 (ref)	1.02 (0.95–1.10)	1.03 (0.95–1.11)	1.10 (1.01–1.20)	1.04 (0.93–1.16)	0.37	1.02 (0.99–1.05)
Dihydrochalcones	1.67 (0.22–5.66)	1.00 (ref)	1.06 (0.98–1.15)	1.10 (1.02–1.20)	1.08 (1.00–1.18)	1.05 (0.97–1.15)	0.60	1.00 (0.99–1.01)
Dihydroflavonols	0.99 (0.00–12.8)	1.00 (ref)	1.07 (0.98–1.18)	1.07 (0.97–1.17)	1.06 (0.96–1.18)	1.08 (0.96–1.22)	0.57	1.00 (1.00–1.01)
Isoflavones	0.03 (0.01–0.78)	1.00 (ref)	1.03 (0.96–1.10)	1.01 (0.94–1.09)	0.99 (0.90–1.07)	0.95 (0.85–1.07)	0.27	1.00 (0.99–1.01)
Phenolic acids	564 (225–1152)	1.00 (ref)	0.98 (0.90–1.06)	1.02 (0.94–1.11)	0.98 (0.90–1.07)	0.97 (0.88–1.06)	0.48	0.98 (0.95–1.02)
Hydroxycinnamic acids	513 (167–1099)	1.00 (ref)	0.93 (0.86–1.01)	0.98 (0.90–1.06)	0.96 (0.88–1.05)	0.96 (0.88–1.06)	0.78	0.99 (0.96–1.02)
Hydroxybenzoics acids	24.1 (4.74–141)	1.00 (ref)	0.95 (0.87–1.02)	0.98 (0.89–1.08)	0.98 (0.89–1.08)	0.96 (0.87–1.06)	0.69	0.99 (0.97–1.01)
Hydroxyphenylacetic acids	0.20 (0.01–0.92)	1.00 (ref)	0.90 (0.83–0.98)	0.98 (0.89–1.08)	0.93 (0.83–1.04)	0.86 (0.76–0.99)	0.07	0.99 (0.98–1.01)
Stilbenes	0.84 (0.04–8.42)	1.00 (ref)	1.02 (0.93–1.11)	1.06 (0.97–1.16)	1.06 (0.96–1.17)	1.07 (0.95–1.20)	0.48	1.01 (1.00–1.02)
Lignans	1.51 (0.90–3.14)	1.00 (ref)	1.10 (1.02–1.19)	1.06 (0.98–1.16)	1.09 (0.98–1.20)	1.02 (0.90–1.15)	0.54	1.00 (0.95–1.05)
Other (poly)phenols	59.0 (23.7–115)							
Alkylphenols	41.2 (4.01–99.0)	1.00 (ref)	0.98 (0.88–1.08)	0.99 (0.89–1.09)	1.01 (0.90–1.12)	1.01 (0.89–1.15)	0.59	1.02 (1.00–1.05)
Tyrosols	3.97 (0.77–24.5)	1.00 (ref)	0.97 (0.89–1.05)	1.04 (0.95–1.13)	1.02 (0.92–1.13)	0.99 (0.86–1.14)	0.75	1.00 (0.99–1.02)
Alkylmethoxyphenols	2.79 (0.64–6.17)	1.00 (ref)	0.96 (0.88–1.05)	1.00 (0.92–1.09)	0.96 (0.88–1.06)	0.93 (0.84–1.03)	0.13	0.99 (0.98–1.01)

Abbreviations: CI, confidence interval; HR, hazard ratio; P10, 10th percentile; P90, 90th percentile. Cox model (model 3) was stratified according to age (5 y) and center, and adjusted for smoking status, physical activity, educational level, marital status, and diabetes prevalence, and alcohol, BMI, total energy, fiber, and vitamin C intakes.

**Table 4:** Hazard Ratios (CI 95%) for prostate cancer death, grade, and stage of total, classes and subclasses of (poly)phenol intakes in the EPIC cohort.

	PCa Grade			PCa Stage		P-Heterogeneity ^2^	**Fatal PCa**
	Low	High	P-Heterogeneity ^1^	Localized	Advanced		Continuous (log2) HR (95% CI)
	Continuous (log2) HR (95% CI)	Continuous (log2) HR (95% CI)		Continuous (log2) HR (95% CI)	Continuous (log2) HR (95% CI)		
Total (poly)phenols	1.01 (0.94–1.08)	1.05 (0.89–1.24)	0.47	0.98 (0.90–1.07)	1.03 (0.91–1.17)	0.56	0.98 (0.90–1.06)
Flavonoids	1.01 (0.97–1.05)	1.02 (0.93–1.12)	0.74	1.01 (0.96–1.06)	1.02 (0.95–1.09)	0.05	0.98 (0.91–1.05)
Total Flavanols	1.01 (0.98–1.05)	1.01 (0.93–1.09)	0.89	1.01 (0.97–1.05)	1.01 (0.95–1.07)	0.06	0.99 (0.95–1.04)
Flavan-3-ol monomers	1.00 (0.98–1.02)	1.00 (0.96–1.05)	0.70	1.00 (0.98–1.03)	0.99 (0.96–1.02)	0.29	1.00 (0.97–1.03)
Proanthocyanidins	1.02 (1.00–1.04)	0.98 (0.94–1.02)	0.12	1.00 (0.99–1.02)	1.02 (0.98–1.07)	0.24	1.00 (0.99–1.02)
Theaflavins	1.01 (1.00–1.01)	1.00 (0.99–1.01)	0.49	1.00 (1.00–1.01)	1.00 (0.99–1.01)	0.45	0.99 (0.92–1.07)
Flavonols	1.00 (0.96–1.04)	1.02 (0.94–1.11)	0.61	1.02 (0.97–1.06)	1.00 (0.94–1.06)	0.25	0.98 (0.93–1.02)
Flavanones	0.99 (0.97–1.01)	0.99 (0.94–1.04)	0.72	0.98 (0.95–1.00)	1.00 (0.96–1.05)	0.72	1.01 (0.97–1.06)
Anthocyanins	1.01 (0.99–1.04)	0.99 (0.94–1.04)	0.34	1.02 (0.99–1.04)	1.03 (0.99–1.08)	0.08	1.02 (0.93–1.11)
Flavones	1.02 (0.98–1.07)	0.99 (0.90–1.09)	0.61	1.02 (0.97–1.07)	1.02 (0.94–1.10)	0.01	0.99 (0.96–1.01)
Dihydrochalcones	1.00 (0.99–1.02)	0.99 (0.96–1.02)	0.31	1.00 (0.98–1.01)	1.02 (0.99–1.04)	0.44	0.99 (0.97–1.01)
Dihydroflavonols	1.01 (1.00–1.02)	0.99 (0.96–1.01)	0.06	1.00 (0.99–1.02)	0.99 (0.98–1.01)	0.34	1.00 (0.97–1.04)
Isoflavones	0.99 (0.98–1.00)	0.96 (0.96–1.02)	0.20	0.99 (0.97–1.01)	1.01 (0.99–1.04)	0.77	0.97 (0.88–1.07)
Phenolic acids	1.00 (0.95–1.05)	1.01 (0.91–1.13)	0.23	0.98 (0.93–1.04)	1.00 (0.92–1.08)	0.23	0.97 (0.90–1.06)
Hydroxycinnamic acids	1.00 (0.96–1.04)	1.00 (0.92–1.11)	0.49	0.99 (0.94–1.04)	0.99 (0.93–1.06)	0.40	0.98 (0.93–1.03)
Hydroxybenzoics acids	0.99 (0.96–1.01)	1.00 (0.95–1.06)	0.74	0.99 (0.96–1.02)	0.98 (0.94–1.03)	0.51	0.99 (0.95–1.03)
Hydroxyphenylacetic acids	1.00 (0.98–1.02)	0.98 (0.94–1.04)	0.44	1.00 (0.98–1.03)	0.99 (0.95–1.04)	0.57	0.98 (0.95–1.02)
Stilbenes	1.01 (1.00–1.03)	0.97 (0.94–1.01)	0.06	1.02 (0.99–1.04)	0.99 (0.96–1.02)	0.16	1.03 (0.87–1.21)
Lignans	0.99 (0.92–1.06)	0.94 (0.79–1.12)	0.53	1.02 (0.94–1.12)	1.02 (0.90–1.15)	0.91	1.04 (0.93–1.15)
Other (poly)phenol classes							
Alkylphenols	1.01 (0.98–1.04)	1.02 (0.95–1.08)	0.54	1.02 (0.98–1.06)	1.04 (0.98–1.10)	0.01	0.98 (0.94–1.02)
Tyrosols	1.03 (0.99–1.06)	0.97 (0.91–1.04)	0.08	1.05 (1.01–1.09)	0.98 (0.93–1.04)	0.69	0.99 (0.95–1.04)
Alkylmethoxyphenols	1.00 (0.98–1.02)	0.99 (0.95–1.04)	0.78	1.00 (0.98–1.02)	0.97 (0.93–1.01)	0.78	0.99 (0.95–1.04)

Abbreviations: CI, confidence interval; HR, hazard ratio; P10, 10th percentile; P90, 90th percentile. Cox model (model 3) was stratified according to age (5 y) and center, and adjusted for smoking status, physical activity, educational level, marital status, and diabetes prevalence, and alcohol, BMI, total energy, fiber, and vitamin C intakes. ^1^ *p*-value for heterogeneity for high- versus low-grade prostate cancer. ^2^ *p*-value for heterogeneity for advanced versus localized prostate cancer.

## Data Availability

For information on how to submit an application for gaining access to EPIC data and/or biospecimens, please follow the instructions at http://epic.iarc.fr/access/index.php (accessed on 9 August 2023).

## Supplementary Materials

The following supporting information can be downloaded at: https://www.mdpi.com/article/10.3390/cancers15164067/s1. Supplementary Table S1: Baseline characteristics of participants according to quintiles of total (poly)phenol intake in the EPIC cohort. Supplementary Table S2: Hazard ratios (CI 95%) for total prostate cancer, according to quartile of intake of total polyphenols, flavonoids, phenolic acids, stilbenes, lignans, and other (poly)phenol classes by body mass index categories in the EPIC study.
