# Supplementary material for: Intake of the Total, Classes, and Subclasses of (Poly)Phenols and Risk of Prostate Cancer: A Prospective Analysis of the EPIC Study
Source: Cancers (Basel). 2023 Aug 11;15(16):4067. doi: 10.3390/cancers15164067 (PMC10452452; doi:10.3390/cancers15164067)
Supplement: Supplementary file 1 [file cancers-15-04067-s001.zip › cancers-2499069-supplementary.pdf]

Table S1. Baseline characteristics of prostate cancer cases without and with grade or stage information in the EPIC cohort.

Baseline characteristics	Grade		Stage	
	**Without data**	**With data**	**Without data**	**With data**
N (%)	3,171 (45.7%)	3,768 (54.3%)	2,764 (39.8%)	4,175 (61.2%)
	Mean (SD)	Mean (SD)	Mean (SD)	Mean (SD)
(Poly)phenol intake (mg/d)	1277.9 (538.6)	1203.4 (509.3)	1242.9 (535.1)	1233.8 (516.9)
Age at recruitment (years)	59.6 (7)	56.9 (6.4)	59.6 (7)	57.1 (6.5)
Total energy intake (kcal)	2437.9 (622.8)	2345.2 (642.3)	2445.8 (620.7)	2349 (641.7)
Fiber intake (mg/d)	23.5 (7.9)	24.5 (8.2)	23.3 (7.9)	24.5 (8.1)
Vitamin C intake (mg/d)	108.9 (56.1)	111.4 (59.8)	109.9 (57.3)	110.5 (58.8)
	N (%)	N (%)	N (%)	N (%)
Smoking status				
Never	996 (31.4%)	1383 (36.7%)	889 (32.2%)	1490 (35.7%)
Former	1381 (43.6%)	1425 (37.8%)	1210 (43.8%)	1596 (38.2%)
Current	755 (23.8%)	938 (24.9%)	634 (22.9%)	1059 (25.4%)
Not specified	39 (1.2%)	22 (0.6%)	31 (1.1%)	30 (0.7%)
Physical activity level				
Inactive	692 (21.8%)	710 (18.8%)	599 (21.7%)	803 (19.2%)
Moderately inactive	1015 (32.0%)	1229 (32.6%)	920 (33.3%)	1324 (31.7%)
Moderately active	719 (22.7%)	935 (24.8%)	620 (22.4%)	1034 (24.8%)
Active	710 (22.4%)	831 (22.1%)	596 (21.6%)	945 (22.6%)
Not specified	35 (1.1%)	63 (1.7%)	29 (1.0%)	69 (1.7%)
Educational level				
None	28 (0.9%)	223 (5.9%)	26 (0.9%)	225 (5.4%)
Primary	1127 (35.5%)	1205 (32.0%)	984 (35.6%)	1348 (32.3%)
Technical/Professional	755 (23.8%)	861 (22.9%)	642 (23.2%)	974 (23.3%)
Secondary	367 (11.6%)	375 (10.0%)	322 (11.6%)	420 (10.1%)
Longer (University)	796 (25.1%)	968 (25.7%)	711 (25.7%)	1053 (25.2%)
Not specified	98 (3.1%)	136 (3.6%)	79 (2.9%)	155 (3.7%)
Marital status				
Single	373 (11.8%)	266 (7.1%)	357 (12.9%)	282 (6.8%)
Together	1705 (53.8%)	1950 (51.8%)	1591 (57.6%)	2064 (49.4%)
Not specified	1093 (34.5%)	1552 (41.2%)	816 (29.5%)	1829 (43.8%)
Diabetes prevalence				
No	2360 (74.4%)	3513 (93.2%)	2074 (75.0%)	3799 (91.0%)
Yes	67 (2.1%)	129 (3.4%)	61 (2.2%)	135 (3.2%)
Not specified	744 (23.5%)	126 (3.3%)	629 (22.8%)	241 (5.8%)
Alcohol intake (g/d)				
0.0	237 (7.5%)	200 (5.3%)	216 (7.8%)	221 (5.3%)
>0.0 - < 5.0	597 (18.8%)	865 (23.0%)	526 (19.0%)	936 (22.4%)
5.0–14.9	991 (31.3%)	964 (25.6%)	874 (31.6%)	1081 (25.9%)
15.0–29.9	688 (21.7%)	761 (20.2%)	610 (22.1%)	839 (20.1%)
≥ 30.0	658 (20.8%)	978 (26.0%)	538 (19.5%)	1098 (26.3%)

Body Mass Index (kg/m²)				
<22.5	389 (12.3%)	331 (8.8%)	349 (12.6%)	371 (8.9%)
≥ 22.5–24.9	871 (27.5%)	875 (23.2%)	777 (28.1%)	969 (23.2%)
≥ 25.0–29.9	1562 (49.3%)	2002 (53.1%)	1349 (48.8%)	2215 (53.1%)
≥ 30.0	349 (11.0%)	560 (14.9%)	289 (10.5%)	620 (14.9%)

Table S2. Hazard ratios (CI 95%) for total prostate cancer, according to quintile of intake of total polyphenols, flavonoids, phenolic acids, stilbenes, lignans, and other (poly)phenol classes by body mass index categories in the EPIC study.

	**BMI < 25**	**BMI 25–29.9**	**BMI ≥ 30**	***P* for interaction**
	**HR (95% CI)**	**HR (95% CI)**	**HR (95% CI)**	
N	48,703	63,710	19,012	
Cases	2,466	3,564	909	
Total (poly)phenols				0.05
Quintile 1	1.00 (ref)	1.00 (ref)	1.00 (ref)	
Quintile 2	1.09 (0.96-1.23)	1.09 (0.98-1.22)	1.11 (0.89-1.38)	
Quintile 3	1.04 (0.91-1.19)	0.98 (0.87-1.10)	1.19 (0.95-1.49)	
Quintile 4	0.93 (0.80-1.08)	1.05 (0.92-1.18)	1.11 (0.87-1.41)	
Quintile 5	0.91 (0.77-1.08)	1.05 (0.91-1.20)	1.17 (0.90-1.53)	
P–trend	0.09	0.72	0.35	
Continuous (log2)	0.94 (0.86-1.03)	1.00 (0.93-1.07)	1.07 (0.94-1.22)	
Flavonoids				0.10
Quintile 1	1.00 (ref)	1.00 (ref)	1.00 (ref)	
Quintile 2	0.97 (0.86-1.09)	0.96 (0.87-1.07)	1.09 (0.88-1.35)	
Quintile 3	0.99 (0.86-1.13)	0.99 (0.89-1.11)	1.10 (0.88-1.38)	
Quintile 4	0.97 (0.84-1.12)	1.03 (0.92-1.16)	1.07 (0.85-1.36)	
Quintile 5	0.84 (0.72-0.99)	0.97 (0.85-1.1)	1.14 (0.89-1.47)	
P–trend	0.05	0.86	0.42	
Continuous (log2)	0.99 (0.94-1.04)	1.01 (0.97-1.06)	1.07 (0.98-1.16)	
Phenolic acids				0.56
Quintile 1	1.00 (ref)	1.00 (ref)	1.00 (ref)	
Quintile 2	0.91 (0.8-1.04)	1.02 (0.91-1.14)	0.98 (0.78-1.21)	
Quintile 3	0.94 (0.82-1.08)	1.09 (0.98-1.22)	0.94 (0.74-1.18)	
Quintile 4	0.93 (0.81-1.07)	1.04 (0.92-1.17)	1.04 (0.82-1.31)	
Quintile 5	0.9 (0.77-1.05)	0.99 (0.87-1.13)	1.06 (0.82-1.38)	
P–trend	0.32	0.72	0.48	
Continuous (log2)	0.97 (0.92-1.03)	0.99 (0.94-1.04)	1.01 (0.93-1.11)	
Stilbenes				0.74
Quintile 1	1.00 (ref)	1.00 (ref)	1.00 (ref)	
Quintile 2	1.08 (0.94-1.24)	0.99 (0.88-1.11)	1.21 (0.95-1.53)	
Quintile 3	1.07 (0.93-1.24)	1.05 (0.93-1.19)	1.22 (0.95-1.57)	
Quintile 4	1.09 (0.92-1.28)	0.99 (0.87-1.14)	1.34 (1.02-1.76)	
Quintile 5	1.13 (0.94-1.37)	1.07 (0.91-1.26)	1.37 (1.00-1.86)	

P–trend	0.42	0.38	0.26	
Continuous (log2)	1.01 (0.99-1.03)	1.00 (0.99-1.02)	1.03 (0.99-1.07)	
Lignans				0.19
Quintile 1	1.00 (ref)	1.00 (ref)	1.00 (ref)	
Quintile 2	1.12 (0.98-1.26)	1.1 (0.99-1.22)	1.07 (0.87-1.33)	
Quintile 3	1.03 (0.90-1.19)	1.1 (0.98-1.24)	1.09 (0.86-1.37)	
Quintile 4	1.02 (0.87-1.21)	1.11 (0.97-1.27)	1.15 (0.88-1.50)	
Quintile 5	0.95 (0.77-1.17)	1.00 (0.84-1.19)	1.21 (0.87-1.66)	
P–trend	0.29	0.44	0.30	
Continuous (log2)	1.01 (0.92-1.10)	0.96 (0.89-1.03)	1.13 (0.98-1.30)	
Other (poly)phenol classes				0.09
Quintile 1	1.00 (ref)	1.00 (ref)	1.00 (ref)	
Quintile 2	0.97 (0.85-1.11)	1.04 (0.93-1.16)	1.04 (0.83-1.30)	
Quintile 3	0.97 (0.84-1.13)	1.03 (0.92-1.16)	1.11 (0.88-1.40)	
Quintile 4	1.08 (0.93-1.26)	1.00 (0.88-1.14)	1.06 (0.82-1.37)	
Quintile 5	1.03 (0.86-1.24)	1.06 (0.92-1.23)	1.07 (0.80-1.43)	
P–trend	0.47	0.53	0.76	
Continuous (log2)	1.02 (0.95-1.09)	1.02 (0.97-1.08)	1.04 (0.93-1.15)	

Abbreviations: CI, confidence interval; HR, hazard ratio.

Cox model was stratified by age (5y) and center, and adjusted for smoking status, physical activity, educational level, marital status, diabetes prevalence, and alcohol, BMI (as continuous) total energy, fiber, and vitamin C intakes.
